# Stakeholder-Based Methods to Develop a Toolkit to Promote Engagement in Assisted Living Safety

**DOI:** 10.1093/geroni/igab046.1331

**Published:** 2021-12-17

**Authors:** Anna Beeber, Ruth Anderson, Matthias Hoben, Stephanie Chamberlain, Victoria Bartoldus, Stephanie Palmertree

**Affiliations:** 1 University of North Carolina at Chapel Hill, UNC Chapel Hill, North Carolina, United States; 2 University of Alberta at Edmonton, Edmonton, Alberta, Canada; 3 University of Alberta, University of Alberta, Alberta, Canada; 4 University of North Carolina at Chapel Hill, Chapel Hill, North Carolina, United States; 5 University of North Carolina at Chapel Hill School of Nursing, Chapel Hill, North Carolina, United States

## Abstract

This presentation provides and overview of a mixed-methods stakeholder engaged study to develop a toolkit to encourage resident and family engagement in the safety of assisted living (AL). This study uses stakeholder-based data and stakeholder engaged processes to adapt existing tools and strategies from other settings to encourage resident and family engagement in the safety of AL. We will improve resident safety in AL by developing an evidence-based tool to implement these engagement tools/strategies in AL. The presentation will outline the theoretical base, the approach for this study, including efforts to recruit and retain stakeholders throughout the study, and stakeholder engaged process to develop the toolkit. The presentation will include challenges and strategies to encourage participation of AL staff, residents, and family caregivers in the study. The presentation will conclude with a discussion of implications for future design and research efforts aiming to impact AL care, policy, and research implementation.

